# High expression of NOLC1 as an independent prognostic factor for survival in patients with colorectal cancer

**DOI:** 10.1007/s00432-023-05297-7

**Published:** 2023-09-05

**Authors:** Zhiwei Sun, Qianshi Zhang, Jinjuan lv, Yuzhu Sun, Zhen Feng, Mengyan Zhang, Feifan Zhang, Cong Xia, Yina Gao, Zhenyu Zhang, Yun-Fei Zuo, Shuang-Yi Ren

**Affiliations:** 1 Department of Gastrointestinal Surgery, The Second Affiliated Hospital of Dalian Medical University, Dalian, 116023 China; 2 Department of Clinical Biochemistry, College of Laboratory Diagnostic Medicine, Dalian Medical University, Dalian, China

**Keywords:** Nucleolar and coiled-body phosphoprotein 1 (NOLC1), Colorectal cancer, Pathways enrichment analyses, Prognostic factors, Prognostic biomarker

## Abstract

**Background:**

As a phosphorylated protein, NOLC1 is mainly located in the nucleus and is highly expressed in a variety of tumors, participating in the regulation of cell proliferation and aging. This study further investigated the role of NOLC1 in colorectal cancer tumors, aiming to provide sufficient scientific evidence for the clinical treatment of colorectal cancer.

**Methods:**

We used TCGA, GEO, TNMplot, GEPIA, and other databases to explore the expression level of NOLC1 in colorectal cancer patients, as well as the correlation between the clinical characteristics of colorectal cancer patients and their expression, and conducted the prognostic analysis. Immunohistofluorescence (IHF) staining verified the analytical results. Subsequently, KEGG and GO enrichment analysis was used to identify the potential molecular mechanism of NOLC1 promoting the occurrence and development of colorectal cancer. The influence of NOLC1 expression on the immune microenvironment of colorectal cancer patients was further investigated using the TIMER database. GDSC database analysis was used to screen out possible anti-colorectal cancer drugs against NOLC1. Finally, we demonstrated the effect of NOLC1 on the activity and migration of colorectal cancer cells by Edu Cell proliferation assay and Wound Healing assay in vitro.

**Results:**

Our results suggest that NOLC1 is overexpressed in colorectal cancer, and that overexpression of NOLC1 is associated with relevant clinical features. NOLC1, as an independent risk factor affecting the prognosis of colorectal cancer patients, can lead to a poor prognosis of colorectal cancer. In addition, NOLC1 may be associated with MCM10, HELLS, NOC3L, and other genes through participating in Wnt signaling pathways and jointly regulate the occurrence and development of colorectal cancer under the influence of the tumor microenvironment and many other influencing factors. Related to NOLC1: Selumetinib, Imatinib, and targeted drugs such as Lapatinib have potential value in the clinical application of colorectal cancer. NOLC1 enhances the proliferation and migration of colorectal cancer cells.

**Conclusions:**

High expression of NOLC1 as an independent prognostic factor for survival in patients with colorectal cancer. NOLC1 enhances the proliferation and migration of colorectal cancer cells. Further studies and clinical trials are needed to confirm the role of NOLC1 in the development and progression of colorectal cancer.

## Introduction

Colorectal cancer is the most common gastrointestinal tumor and one of the most common causes of death among malignant tumors. According to relevant data, the incidence of colorectal cancer in adults under 50 years has been increasing at a rate of 20% since 2003, and its morbidity and mortality rank third among all malignant tumors (Siegel et al. 2020). The treatment principle of colorectal cancer is a comprehensive treatment based on surgical treatment, but the prognosis of advanced patients is still poor, and the 5-year relative survival rate of stage IV colorectal cancer is only 14%. With the development of gene-targeted therapy, it provides a new direction for clinical medication and treatment of colorectal cancer patients {Ohishi, 2023 #100} (Ohishi et al. 2023). Therefore, an in-depth study of the pathogenesis and development of colorectal cancer and the search for more effective detection indicators are key issues to be addressed in the field of colorectal cancer research.

With the further study of the colorectal cancer transcriptome, more and more key driver genes have been discovered. In addition, it plays a crucial role in the proliferation, differentiation and migration of colorectal cancer cells. Nucleolar and coiled-body phosphoprotein 1 (NOLC1) is a type of phosphoprotein consisting of n-terminal and c-terminal domains and a unique central repeat domain, located mainly in the nucleus, gradually attracting people's attention (Chen et al. 2021b). It is highly expressed in a variety of tumors, such as bladder cancer, breast cancer, cholangiocarcinoma, cervical cancer, etc., and participates in the regulation of cell proliferation, aging and other processes. Huaping Huang found that the NOLC1 gene may play an important role in regulating chemotherapy sensitivity of NSCLC by promoting apoptosis and regulating drug-resistant molecules (Huang et al. 2018). Xiaolong Xu et al. reported that CircRNA inhibits DNA damage repair by interacting with parent gene NOLC1, and participates in the regulation of the drug resistance mechanism of breast cancer (Xu et al. 2020). In addition, Wu et al. (2021) reported that RUNt-associated transcription factor 2 (Runx2) promotes metastasis of clear cell carcinoma by down-regulating NOLC1 in renal cell carcinoma and provides a potential clinical therapeutic target. In conclusion, NOLC1 plays a key role in the occurrence and development of various tumors and is expected to play a role as an early tumor diagnostic marker. However, there are few reports on the mechanism of NOLC1 in colorectal cancer.

To further study the role of NOLC1 in colorectal cancer tumors, we conducted NOLC1 expression analysis and survival prognosis analysis based on data from several large online databases, established a survival prediction model, evaluated the relationship between NOLC1 gene expression and clinical features, and analyzed its metabolic pathway through enrichment analysis. The influence of the tumor microenvironment on NOLC1 was determined by the relationship between immune cell infiltration and immune checkpoint. In addition, we further conducted a drug sensitivity analysis.​Finally, we demonstrate the effect of NOLC1 on the proliferation activity and migration of colorectal cancer cells in vitro, aiming to provide a treatment basis for clinical colorectal cancer patients and improve the survival and prognosis time of patients.

## Results

### The different expressions of NOLC1

**Fig. 1:**
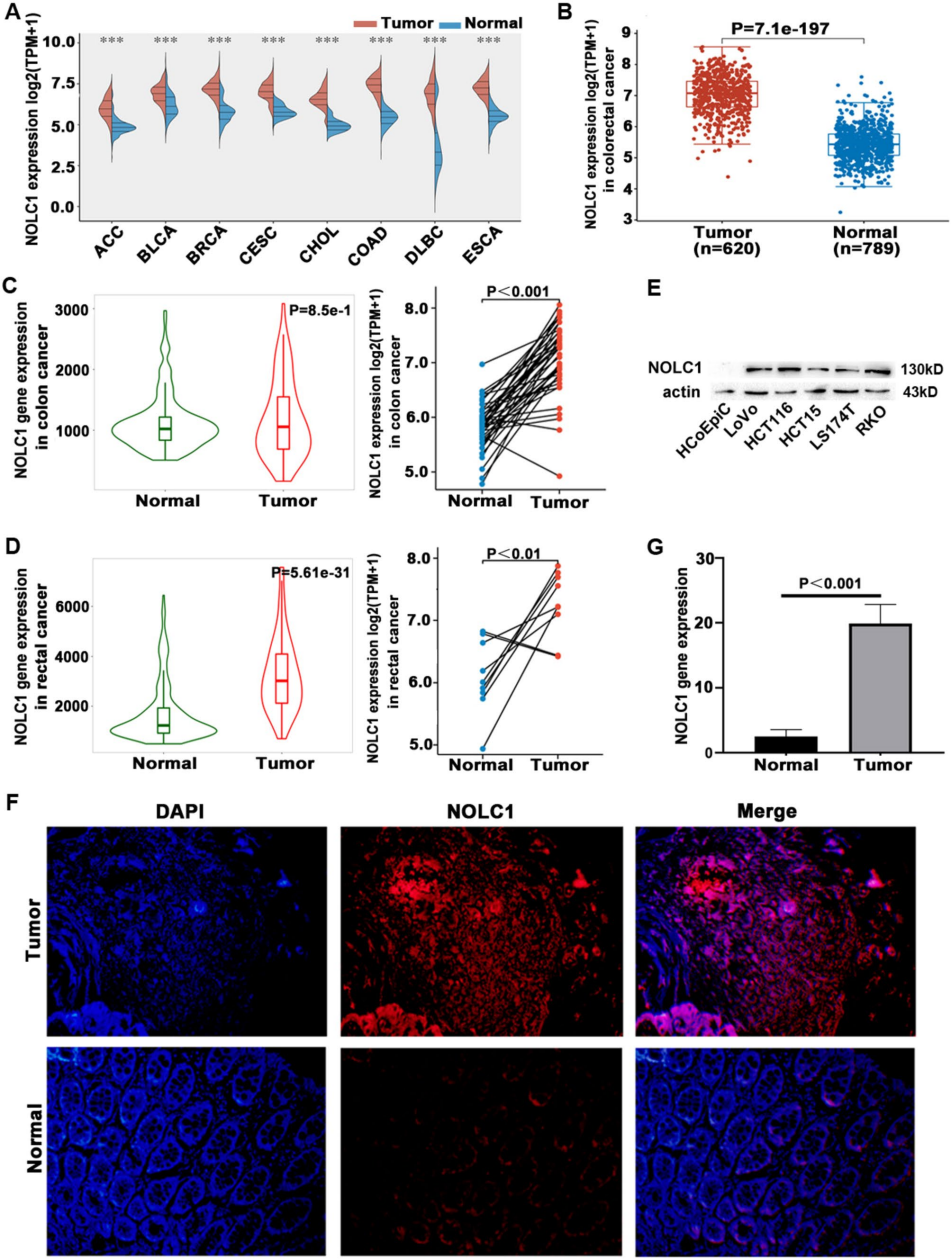
Different mRNA expressions of NOLC1 **a** NOLC1 expression in tumor and normal tissues in TCGA and GTEx pan-cancer data. **b** NOLC1 expression in tumor and normal with colorectal cancer. **c** NOLC1 expression in paired and unpaired colon cancer patients. **d** NOLC1 expression in paired and unpaired patients with rectal cancer. **e** NOLC1 expression in colorectal cancer cell lines using Western blot. **f**, **g** Expression and quantitative analysis of NOLC1 in 16 paired colorectal cancer tissues using immunohistochemistry (IHC) staining

To explore the role of NOLC1 in tumor genesis and development, we evaluated the expression of NOLC1 in generalized carcinoma types using TCGA and GTEx data. The results showed that NOLC1 was highly expressed in ACC, BLCA, BRCA, CESC, and other tumor types compared with normal tissues (Fig. 1a), with statistical significance, and there was a significant difference in colorectal cancer (*P* = 7.1E−197) (Fig. 1b). In addition, data provided by the TNMplot database also showed that NOLC1 expression levels were significantly different in colorectal cancer compared with normal tissues (*P* = 8.5E−1 and 5.6E−31) (Fig. 1c, d). To verify the RNA sequencing results, we used Western blot and immunohistochemistry to confirm the expression of NOLC1 in colorectal cancer cell lines and tissues, and the results confirmed that NOLC1 was overexpressed in colorectal cancer tissues compared with normal colorectal cells and tissues (Fig. 1e–g).

### The prognostic value of NOLC1 in COADREAD

**Fig. 2:**
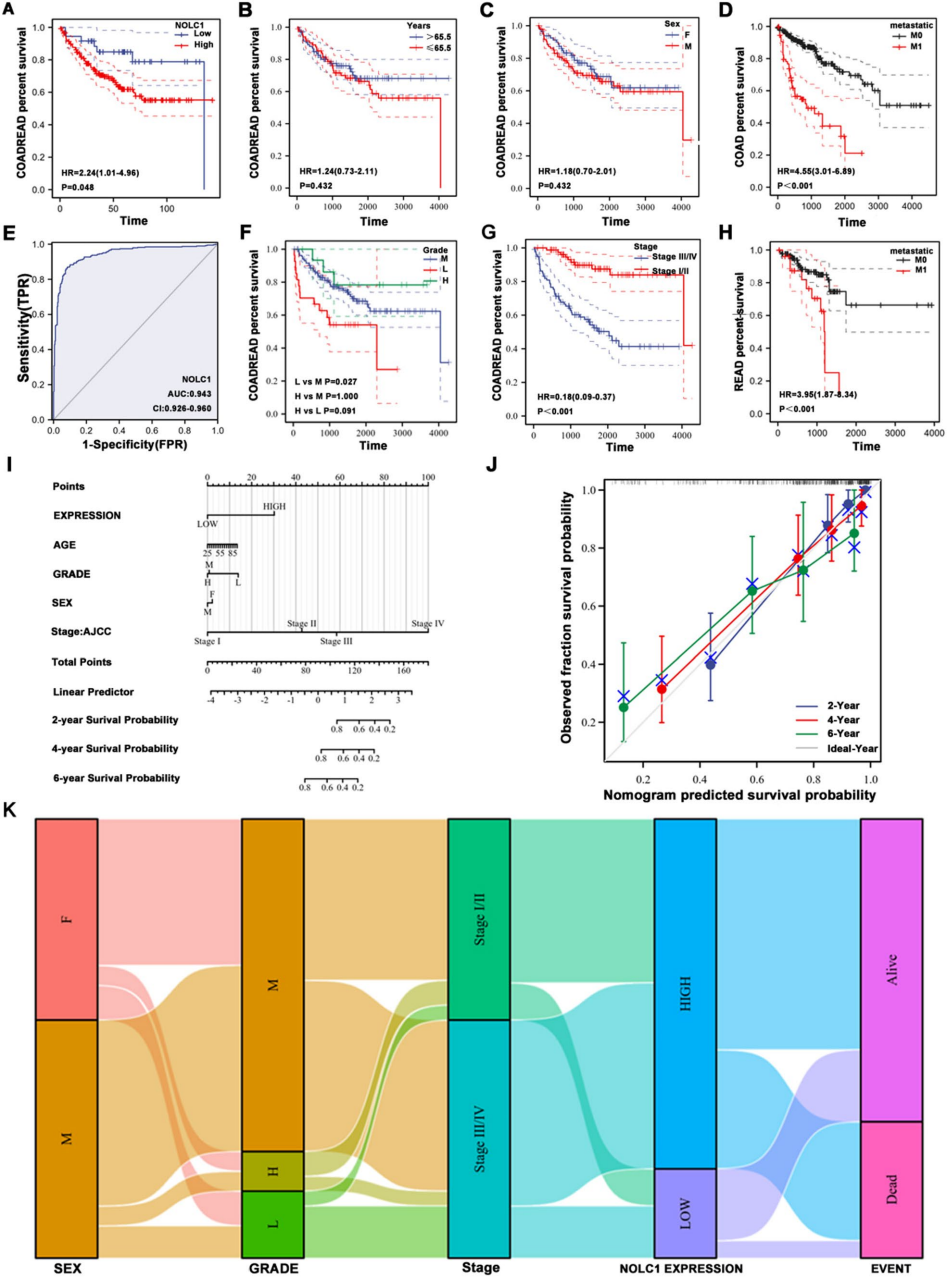
NOLC1 serves as an oncogenic role in COADREAD and high NOLC1 expression predicts poor prognosis **a** Kaplan–Meier curves for high and low expression of NOLC1 subgroup. **b** Kaplan–Meier curves for age subgroup. **c** Kaplan–Meier curves for the gender subgroup. **d**, **h** Kaplan–Meier curves for the metastatic tumor subgroup. **e** ROC curve of diagnosis to distinguish tumor from normal tissue. **f** Kaplan–Meier curves for grade subgroup. **g** Kaplan–Meier curves for stage subgroup. **i** Nomogram model, integrating clinicopathologic factors and NOLC1 level to predict survival probability at 2, 4 and 6 years. **k** Sankey diagram for analyzing visually the relationship between nolc1 expression and clinicopathological factors

From the analysis of the clinical pathology data, we found that the expression level of NOLC1 was correlated with the age of the colorectal cancer patient, post-operative tumor residuals, lymph node invasion, and history of colorectal cancer (Table 1). By K–M survival analysis, we verified the effect of NOLC1 expression and clinicopathological parameters on prognosis. The K–M survival analysis showed that the higher the expression of NOLC1, the worse the overall survival prognosis of colorectal cancer. The difference was statistically significant (*P* = 0.048) (Fig. 2a). We found that age and gender had no effect on overall survival of patients with colorectal cancer (*P* > 0.05) (Fig. 2b, c). We further analyzed the clinical subgroup and found that risk factors such as tumor differentiation degree (*P* = 0.027), distant organ metastasis (*P* < 0.001), and clinicopathological stage (*P* < 0.001) affected the overall survival of colorectal cancer patients (Fig. 2d, f, g, h). In addition, we performed a receiver operating characteristic (ROC) analysis on COADREAD to reveal the diagnostic value of NOLC1 (*P* = 0.943) (Fig. 2e). To further explore the independent risk factors affecting COADREAD patients, univariate and multivariate analyses were performed. The results showed that the pathological clinical stage of tumor (95% CI [0.186 (0.090–0.383)], *P* < 0.001) and NOLC1 (95% CI [0.386 (0.174–0.857)], *P* < 0.019) expression level was an independent risk factor affecting COADREAD patients (Table 2). A nomogram model will be established to predict the 2-year, 4-year, and 6-year survival probability of patients by integrating clinicopathological factors (including age, sex, tumor differentiation, and clinicopathological stage) and NOLC1 expression, and a calibration model will be established to test the reliability of the nomogram model (Fig. 2i, j). At the same time, the effects of different factors on the prognosis of colorectal cancer were visualized by the mulberry map (Fig. 2k).

**Table 1:** Clinical characteristics of the colorectal cancer patients

Characteristic	Low expression of NOLC1	High expression of NOLC1	p
Age, *n* (%)			0.021
≤ 65	123 (19.1%)	153 (23.8%)	
> 65	199 (30.9%)	169 (26.2%)	
Age, median (IQR)	69 (59, 77)	67 (57, 75)	0.027
Gender, *n* (%)			0.874
Female	152 (23.6%)	149 (23.1%)	
Male	170 (26.4%)	173 (26.9%)	
BMI, *n* (%)			0.994
< 25	45 (13.7%)	62 (18.8%)	
≥ 25	95 (28.9%)	127 (38.6%)	
N2	62 (9.7%)	57 (8.9%)	
M stage, *n* (%)			0.324
M0	242 (42.9%)	233 (41.3%)	
M1	51 (9%)	38 (6.7%)	
Pathologic stage, *n* (%)			0.325
Stage I	51 (8.2%)	60 (9.6%)	
Stage II	119 (19.1%)	119 (19.1%)	
Stage III	93 (14.9%)	91 (14.6%)	
Stage IV	53 (8.5%)	37 (5.9%)	
Residual tumor, *n* (%)			0.035
R0	240 (47.1%)	228 (44.7%)	
R1	5 (1%)	1 (0.2%)	
R2	25 (4.9%)	11 (2.2%)	
Perineural invasion, *n* (%)			0.891
No	62 (26.4%)	113 (48.1%)	
Yes	20 (8.5%)	40 (17%)	
Lymphatic invasion, *n* (%)			0.002
No	160 (27.5%)	190 (32.6%)	
Yes	138 (23.7%)	94 (16.2%)	
History of colon polyps, *n* (%)			< 0.001
No	175 (31.5%)	202 (36.4%)	
Yes	112 (20.2%)	66 (11.9%)	
OS event, *n* (%)			0.325
Alive	252 (39.1%)	263 (40.8%)	
Dead	70 (10.9%)	59 (9.2%)	
PFI event, *n* (%)			0.071
Alive	229 (35.6%)	250 (38.8%)	
Dead	93 (14.4%)	72 (11.2%)

**Table 2:** Univariate and multivariate Cox regression analyses of clinical characteristics associated with overall survival

Characteristics	Total (*N*)	Univariate analysis		Multivariate analysis	
		Hazard ratio (95% CI)	*P* value	Hazard ratio (95% CI)	*P* value
Stage	177				
Stage III/IV	96	Reference			
Stage I/II	81	0.183 (0.089–0.374)	< 0.001	0.186 (0.090–0.383)	< 0.001
NOLC1 expression	177				
High	141	Reference			
Low	36	0.447 (0.202–0.992)	0.048	0.386 (0.174–0.857)	0.019
Sex	177				
F	81	Reference			
M	96	1.187 (0.692–2.036)	0.534		
Grade	177				
M	134	Reference			
L	27	2.267 (1.205–4.264)	0.011	1.682 (0.892–3.174)	0.108
H	16	0.568 (0.175–1.844)	0.347	0.582 (0.178–1.898)	0.369
Age	177				
> 65.5	94	Reference			
≤ 65.5	83	1.238 (0.727–2.107)	0.432		

### Correlation and enrichment analyses of NOLC1 in COADREAD

**Fig. 3:**
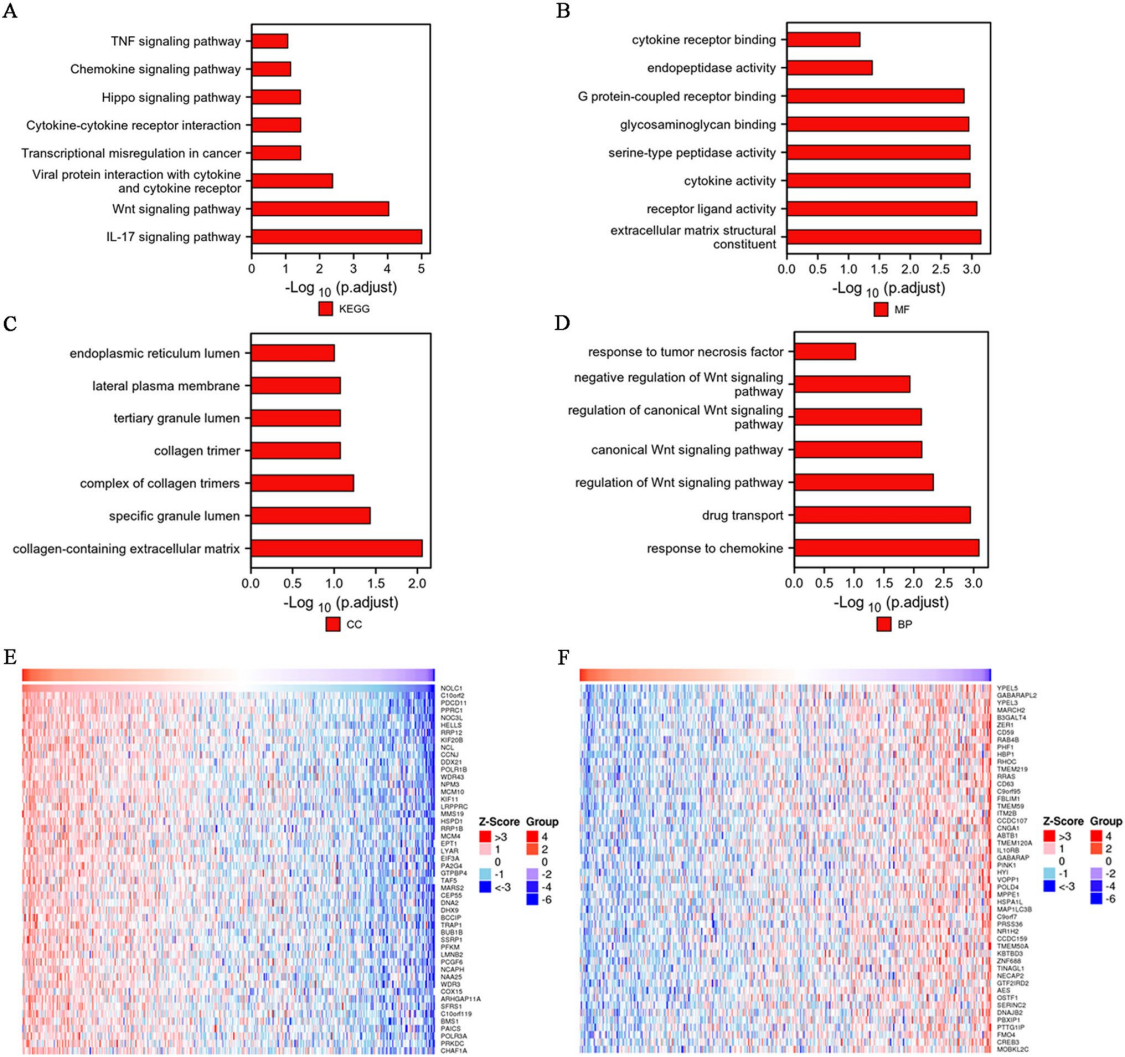
KEGG and GO pathways enriched analyses results and association between NOLC1 expression **a** Significant KEGG pathways of associated with NOLC1. **b–d** Gene Ontology terms related to NOLC1, including molecular function (MF), cell component (CC), and biological processes (BP). **e** Top 50 genes most positively associated with NOLC1 are shown in a heatmap. **f** Top 50 genes most negatively associated with FCGBP are shown in a heatmap

We performed a correlation analysis between NOLC1 and other genes in COADREAD using the TCGA database. In addition, since NOLC1 was found to be related to the progression and prognosis of COADREAD, we further predicted the biological function of NOLC1 in COADREAD and its related regulatory pathways using GO function and KEGG pathway analysis. KEGG results showed that significant signaling pathways associated with NOLC1 overexpression included the IL-17 signaling pathway, the Wnt signaling pathway, and the interaction between viral proteins and cytokines and receptors (Fig. 3a). By analyzing its molecular functions, we found that NOLC1 is not only involved in the structure of the extracellular matrix but also in the regulation of receptor–ligand activity and cytokine activity (Fig. 3b). NOLC1 is also involved in the formation of a complex of collagen trimers, specific granule lumen, and collagen-containing extracellular matrix (Fig. 3c). More importantly, NOLC1 is involved in tumor genesis and development through chemokine responses, drug transport, and positive and negative regulation of Wnt signaling pathways (Fig. 3d). Genes positively correlated with NOLC1 include NOLC1, MCM10, HELLS, and NOC3L, etc. (Fig. 3e), while genes negatively correlated with NOLC1 include ZER1, YPEL3, PHF1, etc. (Fig. 3f).

### NOLC1 cellular localization and genetic alteration in patients with COADREAD

**Fig. 4:**
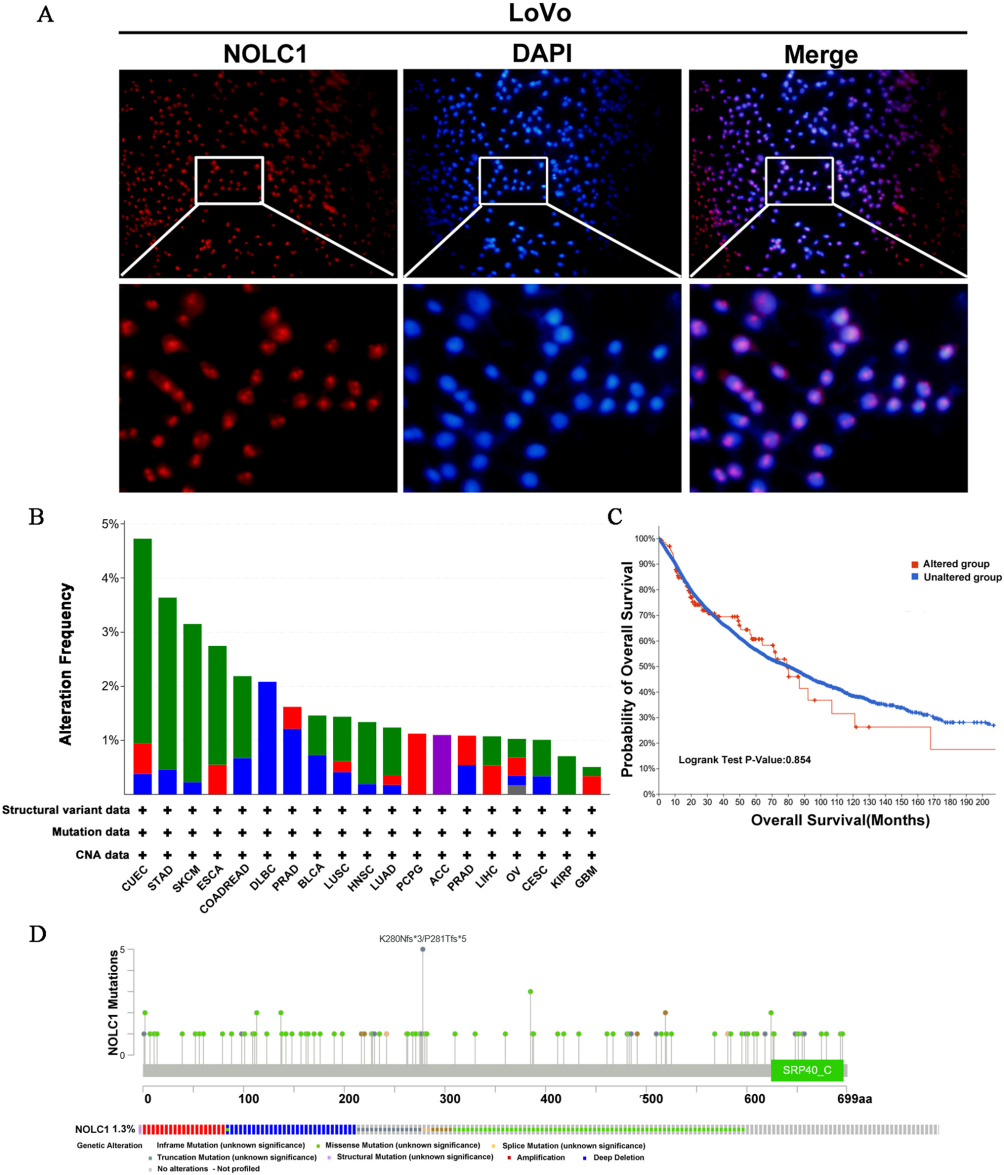
NOLC1 cellular localization and genetic changes in COADREAD patients **a** IF assay were performed to NOLC1 colorectal cancer cell localization. **b** Mutation frequency of NOLC1 in generalized carcinoma analysis. **c** Effect of NOLC1 gene mutation on overall survival was investigated based on TCGA RNA-seq. **d** Mutation details of NOLC1 in each individual sample

Immunofluorescence experiments showed that NOLC1 is mostly located in the nucleus (Fig. 4a). It participates in the coding of nucleoli and ribosomal proteins, which play an important role in transcription and translation. We further studied NOLC1 mutations using the cBioPortal database. In addition, it turns out, in the pan-cancer analysis, NOLC1 was mutated in a variety of cancers, such as endometrial cancer, gastric cancer, skin melanoma, etc., with only 2.19% (13/594) of the genetic changes in colorectal cancer (Fig. 4b). Patients with mutations and log-rank tests showed no significant difference in OS (*P* = 0.854) between patients with mutations and those without alterations (Fig. 4c). In a study from TCGA PanCancer Atlas Studies, the percentage of NOLC1 gene changes was only 1.3% (Fig. 4d), which mainly included amplification mutations, deep deletion mutations, truncation mutations, and missense mutations.

### Correlation between NOLC1 and the immune infiltration of COADREAD

**Fig. 5:**
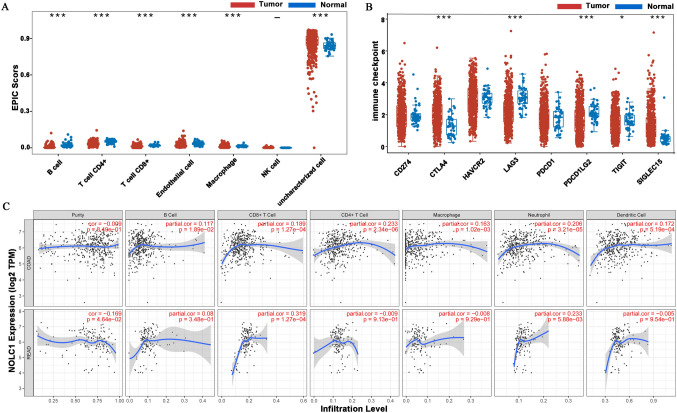
Correlation between NOLC1 expression and immune cell infiltration in the tumor microenvironment **a**, **c** Relationships between NOLC1 expression levels and immune cell infiltration in colorectal cancer. **b** Relationships between NOLC1 expression levels and immune-checkpoints

As an important factor in the process of tumor genesis and development, the tumor microenvironment also plays a huge role in affecting the survival rate of tumor patients (Zhang et al. 2019). We further explored the relationship between NOLC1 mRNA expression and immune cell infiltration by immunoscoring. The results showed that NOLC1 expression was associated with B cells, CD8+ T cells, CD4+ T cells, endothelial cells, macrophages, uncharacterized cells, neutrophils, and dendritic cells (Fig. 5a). At the same time, we use the TIMER1 database (https://cistrome.shinyapps.io/timer/) to further validate our results. In colon cancer, the expression level of NOLC1 was similar to that of B cells (partial COR = 0.117), CD8+ T cells (partial COR = 0.189), CD4+ T cells (partial COR = 0.233), and macrophages (partial COR = 0.163), neutrophils (partial COR = 0.206), and dendritic cells (partial COR = 0.172), which were positively correlated. In rectal cancer, there was a positive correlation between B cells (partial COR = 0.08), CD8+ T cells (partial COR = 0.319) and neutrophils (partial COR = 0.233) (Fig. 5c). In addition, the relationship between NOLC1 and CD274, PDCD1, and PDCD1LG2, which are important immune checkpoints for tumor immune escape, was also explored. We found that the expression levels of CTLA4, LAG3, PDCD1LG2, SIGLEC15, and NOLC1 were significant (Fig. 5b).

### Potential drugs for COADREAD patients

**Fig. 6:**
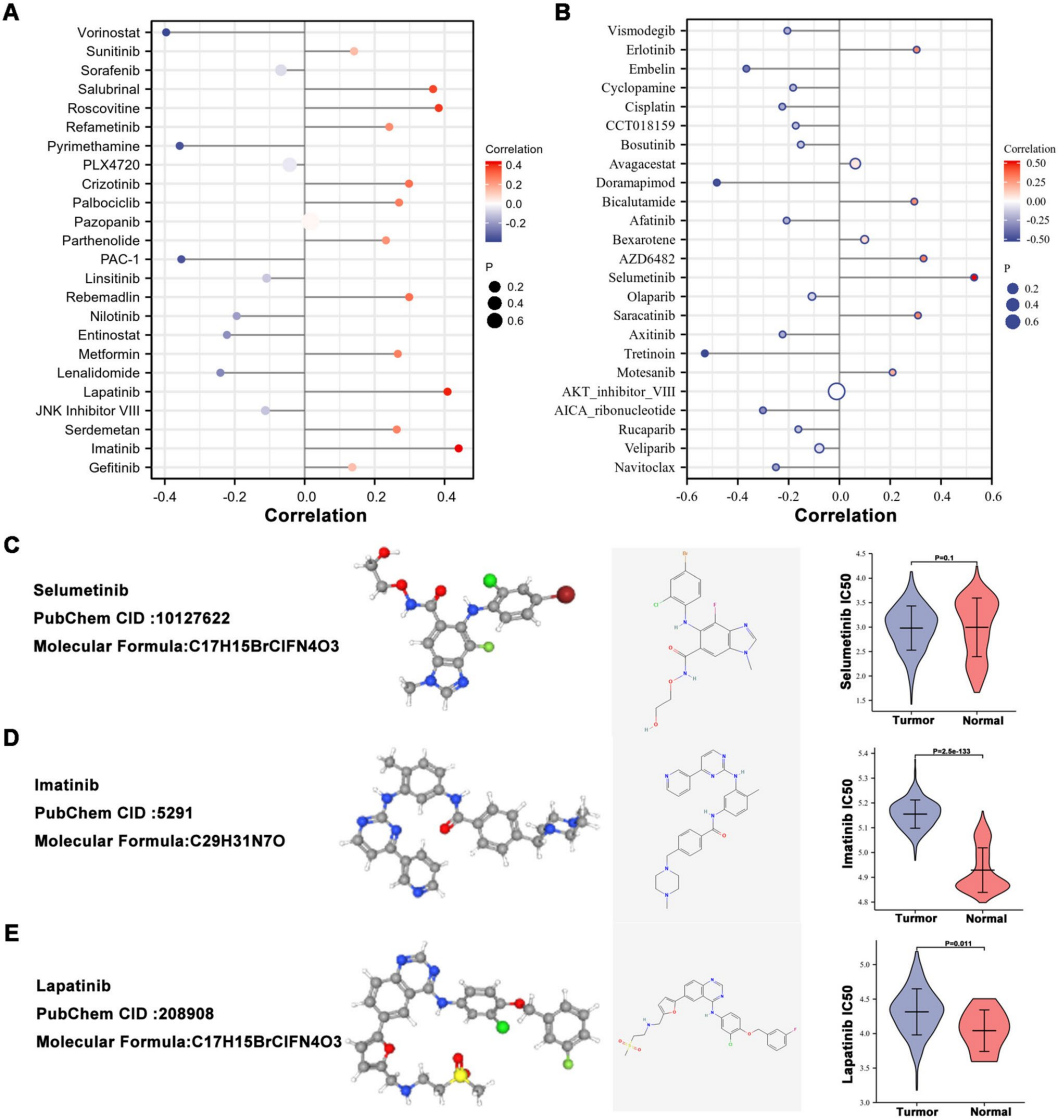
Potential drugs for COADREAD patients **a**, **b** Relationship between NOLC1 expression level and potential targeted drugs. **c**–**e** 2D, 3D structures and IC50 values of drugs targeting NOLC1 in colorectal cancer: Selumetinib; Imatinib; Lapatinib

To identify potential therapeutic targets for colorectal cancer and improve its prognosis, we analyzed the relationship between NOLC1 expression level and drugs based on the GDSC database, and the corresponding parameters are shown (Fig. 6a, b). Three drugs with the strongest correlations were selected, which were Selumetinib (COR = 0.531), Imatinib (COR = 0.44), and Lapatinib (COR = 0.408). Corresponding parameters, the two-dimensional and three-dimensional structures are shown (Fig. 6c–e). We further used GDSC to predict the therapeutic response of high NOLC1 expression levels to drugs.

### NOLC1 enhances the proliferation and migration of colorectal cancer cells

**Fig. 7:**
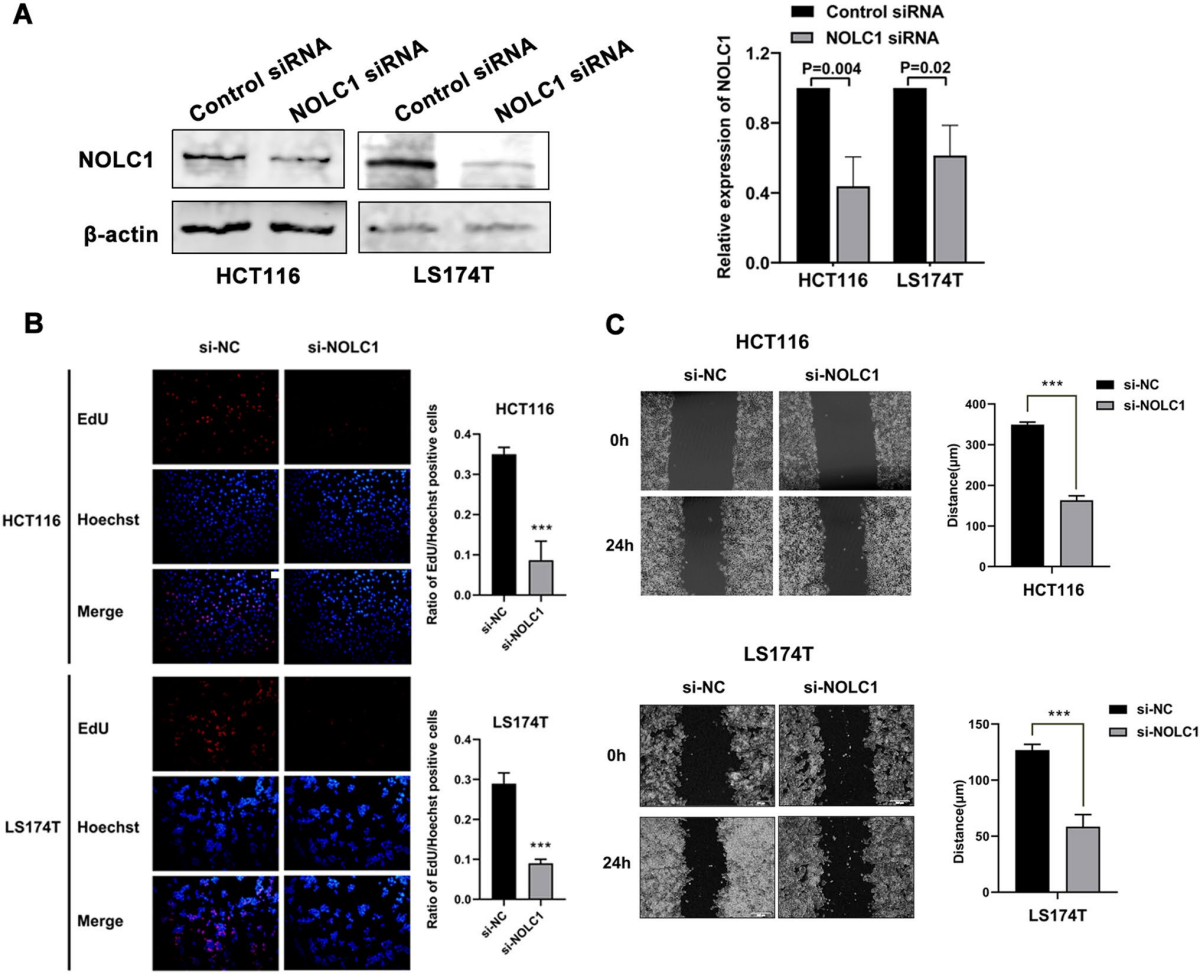
NOLC1 enhances the proliferation and migration of colorectal cancer cells **a** Western Blot analysis showed that the expression of NOLC1 was significantly decreased in colorectal cancer cells after transfection. **b** Cell proliferation assays have shown that down-regulation of NOLC1 can inhibit cell viability in the HCT116 and LS174T cell lines. **c** Wound healing assay showed that down-regulation of NOLC1 was able to inhibit migration in the HCT116 and LS174T cell lines

To investigate whether NOLC1 affects the biological function of colorectal cancer cells, we used EdU assay to measure the proliferation capacity of HCT116 and LS174T cells. The results showed that the expression of NOLC1 was significantly decreased in colorectal cancer cells after transfection (Fig. 7a). In addition, the results show a statistically significant reduction in the ratio of EdU+ to Hoechst33342 stained cells after NOLC1 knockdown. These results suggest that NOLC1 knockdown regulation significantly reduces colorectal cancer cell proliferation in HCT116 and LS174T (Fig. 7b). In addition, we verified the effect of NOLC1 expression on the migration capacity of colorectal cancer cells. It was shown that the migration distance of HCT116 and LS174T cells was significantly shortened after the suppression of NOLC1 expression (Fig. 7c). These results suggest that down-regulation of NOLC1 can significantly inhibit the proliferation and migration of colorectal cancer cells.

## Materials and methods

### Data source and analysis of differential expressions of NOLC1

TCGA (The Cancer Genome Atlas): this database (https://portal.gdc.cancer.gov/) has collected multiple data of more than 20,000 samples of 33 kinds of cancer and generated over 2.5 petabytes of genomic, epigenomic, transcriptomic, and proteomic data. In addition, it provides us with data on cancer-related RNA sequences, clinical, and survival data of tumor pan-cancer. TNMplot provides a comprehensive analysis of RNA sequence expression and Gene chip data for a selected gene in a selected tissue type (https://www.tnmplot.com/). We determined the differential expression of NOLC1 in normal and tumor colorectal tissues.

### Cell culture

Colorectal cancer cell lines HCT-116 and HCT15 were cultured with RPMI-1640 medium containing 10% fetal bovine serum. Colon cancer cell lines LS174T, LoVo, HcoEpic, and RKO were cultured in DMEM medium containing fetal bovine serum. The culture environment was a 37 °C incubator containing 5% CO_2_ and maintaining a certain humidity. All cell lines were purchased from the Institute of Biochemistry and Cell Biology of the Chinese Academy of Sciences (Shanghai, China).

### Western blotting

The cells were denaturated in SDS buffer to obtain total protein, and the protein concentration was determined by the BCA method (Beijing Solaribo Science and Technology). Total protein was separated by the 12% SDS–PAGE gel and transferred onto Millipore PVDF. In addition, sealed with 5% skim milk for 2 h. The PVDF membrane was incubated overnight at 4 °C with rabbit antibodies against NOLC1 (1:1000, Proteintech Group), β-actin (1:1000, Beijing Zhongshan Biotechnology Co., Ltd). After washing, the membrane was incubated with a secondary antibody (Beijing Solaribo Science and Technology) at 37 °C for h.

### Correlations between NOLC1 and clinicopathology or survival in COADREAD

Survival information and clinical data for NOLC1 expression for each colorectal cancer sample were obtained from TCGA (https://portal.gdc.com). Clinical data included age, gender, clinical stage, residual tumor (with tumor or tumor-free), perineural invasion, lymphatic invasion, and history of colon polyps. For Kaplan–Meier curves, *P* values and hazard ratios (HR) with a 95% confidence interval (CI) were generated by log-rank tests and univariate Cox proportional hazards regression. All the analytical methods and R packages were performed using R software version v4.0.3. *P* < 0.05 was considered statistically significant.

### The prognostic factors of NOLC1 in COADREAD

Survival information was selected from the GEO database for each sample of colorectal cancer patients. Then, we selected age, gender, distant metastasis, degree of tumor differentiation, stage and other clinical indicators to evaluate the factors affecting the survival and prognosis of colorectal cancer patients, and selected the median NOLC1 expression as the threshold to explore the influence of NOLC1 expression on the survival and prognosis of colorectal cancer patients through the two-classification method. Kaplan–Meier (K–M) and log-rank tests were applied for survival analysis of colorectal cancer (*P* < 0.05) and survival curves were performed through the R packages “survminer” and “survival.” Univariate and multivariate cox regression analysis was performed to assess NOLC1 and major clinical and prognostic factors to build the nomogram. Using R software (version 3.6.3) with the packages “rms,” to make a nomogram predicting the 2-year, 3-year, and 6-year overall survival of the high-expressed of NOLC1 with colorectal cancer. In addition, we establish the calibration curve of the prediction model. The “GGalluvial” R package was used for the Mulberry map.

### Pathways enrichment analyses of NOLC1 in COADREAD

LinkedOmics (http://www.linkedomics.org/login.php) provides a unique platform for biologists and clinicians to access, analyze, and compare cancer multi-omics data within and across tumor types. To further investigate the tumor-related mechanisms of NOLC1, we performed gene set enrichment analysis in the linkedOmics database. In addition, some tumor-related genetic correlation analysis.

### Genetic alteration in patients with COADREAD

The genomic mutation of NOLC1 was analyzed using the data set of cBioPortal (www.cbioportal.org). In the “Quick selection” section, “TCGA Pan-cancer Map study” was selected, and “NOLC1” was input to query the genetic change characteristics of NOLC1. The Kaplan–Meier curve was established, and survival analysis was performed to explore the relationship between NOLC1 gene variation and prognosis, and the log-rank test was performed, and *P* < 0.05 was considered statistically significant.

### Immunohistofluorescence (IHF) staining

Sixteen pairs of paired colorectal cancer and paired normal adjacent tissue samples were collected from Dalian Medical University and cryopreserved in liquid nitrogen. The surgical specimens of colorectal cancer patients were treated with paraffin embedding, sectioning, and dewaxing, and trypsin (trypsin: PBS = 1:2) was added. Antigen repair was performed and incubated at 37 °C for 30 min. Wash three times for 3 min each with PBS buffer. The 10% normal goat serum (diluted with PBS) was closed and incubated at room temperature for 10 min. Add rabbit [NOLC1 (1:1000, Proteintech Group)] working solution overnight at 4 °C. Biotin-labeled secondary antibody was dropped and incubated at room temperature or 37 °C for 1.5 h. DAPI staining was performed for 15 min, and the slices were sealed for microscopic observation.

### Screening of potential drugs associated with NOLC1 gene

We predicted the chemotherapeutic response for each sample based on the largest publicly available pharmacogenomics database [the Genomics of Drug Sensitivity in Cancer (GDSC), https://www.cancerrxgene.org/]. The prediction process was implemented by the R package (version 4.0.3) “pRRophetic”, where the samples’ half-maximal inhibitory concentration (IC50) was estimated by ridge regression and the prediction accuracy. In addition, Pubch Database (https://pubmed.ncbi.nlm.nih.gov/) is used to query the drug ID, 2D, 3D structure, and other related information.

### Immune-checkpoint analysis

We through the TIMER 1.0 online website (https://cistrome.shinyapps.io/timer/), explore the relationship of NOLC1 and tumor microenvironment, further evidence of NOLC1 expression and correlation immunity infiltration. The corresponding results can be obtained by inputting target genes, tumor types and immune cells through the “gene” plate. CTLA4, CD274, and PDCD1 were selected to be immune-checkpoint–relevant transcripts and the expression values of these three genes were extracted. All the above analysis methods and R packages were implemented by the R Foundation for Statistical Computing (2020) version 4.0.3 and software packages ggplot2 and pheatmap to assess the co-expression of NOLC1 with these immune-checkpoints.

### Cell transfection

A day before transfection, cells of appropriate density were inoculated and evenly dispersed into 12-well plates. After 24 h, the cells are 60–80% fused. Gently mix 100 μl of serum-free medium with 4 μl of GP-Transfect-Mate transfection reagent and incubate at room temperature for 5 min. Mix another 100 μl of serum-free medium with 4 μl of NOLC1 siRNA and let it stand at room temperature for 5 min. The GP-transfection-Mate reagent medium mixture was added to the NOLC1 siRNA medium mixture, and the mixture was allowed to stand at room temperature for 20 min. Add 800 μl of serum-free medium to each well. Add the infusion mixture to each hole and gently shake the dish to evenly distribute. Place the culture plate in an incubator at 37 °C and replace it with fresh and complete culture medium after 6 h.

### Cell proliferation assay

After digestion, the transfected cells were seeded in 24-well plates overnight and returned to normal. Configure a working solution for 2 × EdU. 250ul of EdU working solution preheated at 37 °C was added to each well, mixed, and incubated for 2 h. Add 250 μl of 4% paraformaldehyde to each well and hold at room temperature for 15 min. Add 250 μl of PBS containing 3% BSA to each well and wash three times, each time for 5 min. Discard the washing solution, add 250 μl of PBS containing 0.3% tritonx-100 to each well, and leave at room temperature for 15 min. Click reaction solution should be configured and used within 15 min of configuration. Add 100 μl of Click reaction solution to each well, gently shake and mix to evenly cover the sample and incubate at room temperature for 30 min in the dark. Hoechst 33342 was diluted in PBS at a ratio of 1:1000 and stored away from light. 250 μl 1 × Hoechst 33342 was added to each well and incubated for 10 min at room temperature in the dark. Wash with the wash liquid three times, each time for 5 min. Photographs were taken under a fluorescence microscope.

### Wound-healing assay

The logarithmic growth HCT116 and LS174T cells were digested and centrifuged, and after counting with a counter plate, the cells were added to the well plate according to the desired cell density, gently shaken to evenly distribute the cells in the well plate, and incubated at 37 °C containing 5% CO_2_. On the following day, when the cells have grown sufficiently, the lines are marked with a sterile spear pointing perpendicular to the horizontal line at the bottom of the trap plate. The cells were washed with sterile PBS to remove floating cells. 1 ml of serum-free medium was added to each well at the same time. They were observed and photographed at 0 h and 24 h using an inverted phase contrast microscope. The abrasion widths at 0 h and 24 h were calculated using the software Image J, and statistical analysis was performed.

### Statistical analysis

The statistical analysis for this study was calculated automatically from the above online database. *P* values < 0.05 or log-rank *P* values < 0.05 was considered statistically significant. R software (version 4.0.3) was used for statistical analysis.

## Discussion

Colorectal cancer, as one of the most common gastrointestinal tumors, has the characteristics of high morbidity and mortality, seriously endangering the health of people all over the world. At present, the pathogenesis of colorectal cancer is still unclear, and research on its occurrence and development mechanism is also in the preliminary exploration stage. Therefore, it is of vital importance to find highly sensitive tumor markers for the early screening and diagnosis of colorectal cancer patients. With the further study of tumors, more and more reports have proved that NOLC1, as a protein-coding gene (nucleolar and curular phosphoprotein 1), has a regulatory role in the occurrence and development of various tumors. Fuwen Yuan et al. also found that highly expressed NOLC1 promotes cell senescence and inhibits cell proliferation in HCC and plays a certain role in cell senescence through the CSIG–NOLC1–RRNA pathway (Yuan et al. 2017). Kong et al. (2021) also reported that NOLC1 over-expression is considered to be an independent adverse factor affecting the overall survival of esophageal cancer patients and can participate in the invasion and protein expression of esophageal cancer through the PI3K/AKT pathway. However, the significance of NOLC1 expression in the prognosis of colorectal cancer remains unclear. Further analysis was conducted to further explore the role of NOLC1 in colorectal cancer.

In this study, we first used The Cancer Genome Atlas (TCGA) data to conduct a pan-cancer analysis on NOLC1 expression and found that NOLC1 expression was elevated in colorectal cancer, esophageal cancer, bile duct cancer, and other tumor tissues. Subsequently, the expression of NOLC1 in colorectal cancer was verified by the TNMplot database. Meanwhile, Western Blotting, and IHC staining experiments were performed, and the results also showed that NOLC1 expression was significantly increased in colorectal cancer cells and tissues. The Kaplan–Meier survival analysis, the ROC curve, and univariate and multivariate analysis again confirmed that the higher the NOLC1 scale in colorectal cancer, the worse the survival prognosis and was an independent risk factor affecting the prognosis of colorectal cancer patients. We also established a survival prediction model related to NOLC1 expression to predict the 2-year, 4-year, and 6-year survival rates of colorectal cancer, providing a basis for clinical diagnosis and treatment improvement. These results suggest that NOLC1 can be used as a potential biomarker to predict the prognosis of colorectal cancer.

The specific mechanism of NOLC1 regulation of colorectal cancer is still unclear. We conducted an enrichment pathway analysis to further explore the potential role of NOLC1 in the progression of colorectal cancer. The results showed that NOLC1 was significantly enriched in the IL-17 signaling pathway, the Wnt signaling pathway, and the transcriptional dysregulation pathway in tumors. There is increasing evidence that these signaling pathways play an important role in the genesis and development of tumors. Xun Lin et al. reported that the IL-17 receptor (IL-17RA) is expressed in multiple intestinal cell types and is involved in host defense and inflammatory bowel disease by coordinating multicellular immune responses (Lin et al. 2022). Colorectal cancer (CRC) can be activated by mutations in the typical WNT/β-catenin signaling pathway, but inhibition of the WNT pathway can lead to significant target organ toxicity. Hinze L et al. reported that inhibition of GSK3-dependent protein degradation can be used in the treatment of colorectal cancer and reduce drug toxicity (Hinze et al. 2020). It has also been reported that the wrong regulation of the Wnt signaling pathway is associated with a variety of tumors, such as colon cancer and melanoma (Alves-Guerra et al. 2007). Therefore, NOLC1 may be involved in the regulation of colorectal cancer through the above-mentioned pathways. In addition, GO analysis showed that NOLC1 may also be involved in the formation of the extracellular matrix, regulating receptor activity, cytokine activity, drug transport, and other functions. In addition, we further used the LinkedOmics network analysis tool to explore the co-expression of the NOLC1 gene in an attempt to find the potential genes affecting the occurrence and development of colorectal cancer. We found that NOLC1 was positively correlated with the expression of MCM10, HELLS, and NOC3L genes. Tian J et al. reported that abnormal overexpression of MCM10 induces excessive DNA replication, leading to genomic instability and promoting the proliferation and metastasis of esophageal cancer cells in vitro and in vivo (Tian et al. 2021). Schuller et al. (2022) also found that inactivation of the tumor suppressor gene P53 can lead to overexpression of the HELLS gene in HCC patients, thus affecting the prognosis of HCC. Therefore, we hypothesized that NOLC1 plays an indispensable role in the regulation of the occurrence and development of colorectal cancer, and it may promote the invasion and metastasis of colorectal cancer together with MCM10, HELLS, and Noc3L-related genes.

The proliferation, differentiation, and invasion of tumors are the result of the interaction of multiple genes and the co-regulation of the immune microenvironment. The immune microenvironment is an important factor affecting the occurrence and development of tumors, among which immune cell infiltration also plays an important role (Wei et al. 2022; Sun et al. 2022). In this study, NOLC1 expression was positively correlated with B cells, CD8+ T cells, and neutrophils. Relevant literature has reported that in hepatocellular carcinoma, Treg cells and neutrophils have a high level of immune infiltration, while their OS and DFS are relatively low (Schoenberg et al. 2021). It has also been found that the DIRAS2 gene reduces the overall survival of colorectal cancer patients by blocking the enhancer signaling pathway that activates nuclear factor B (Ying et al. 2022). In addition, there is increasing evidence that immune checkpoints play an important role in the progression of colorectal cancer (Ma et al. 2021b). We further evaluated the relationship between NOLC1 and immune checkpoints. The results showed that in colorectal cancer, the high expression of NOLC1 was closely related to multiple immune checkpoints, such as LAG3, CTLA-4, and PDCD1LG2, leading to immune tolerance or escape of tumor cells. Therefore, immune checkpoint blocking therapy offers a broad therapeutic prospect for patients with high expression of NOLC1 in advanced colorectal cancer (Msaouel et al. 2021; Lelliott et al. 2021; Chen et al. 2021a).

The treatment of colorectal cancer is a comprehensive treatment based on surgery, but the prognosis of patients with advanced colorectal cancer is often poor. Capecitabine oxaliplatin (XELOX) combination therapy has been used by the National Comprehensive Cancer Network (NCCN) as first-line chemotherapy for patients with metastatic colorectal cancer (Benson et al. 2021). However, the peripheral neurotoxicity of oxaliplatin is difficult to tolerate in some patients. Survival time for patients with advanced colorectal cancer has improved significantly with the widespread use of targeted drugs in the clinic. In addition, it reduces nausea, vomiting, neurotoxicity and other side effects caused by conventional chemotherapy. Therefore, there is an urgent need to develop new targeted drugs for the treatment of advanced colorectal cancer. We analyzed the relationship between NOLC1 expression level and drugs based on the GDSC database. Potential drugs with potential clinical application value were screened out. Among them, we selected three small-molecule drugs with the strongest correlation. Although these drugs are not the first choice for colorectal cancer, numerous previous studies have shown that they have different clinical effects on different tumor types. Grasso et al. reported concentration-dependent effects of Selumetinib in colorectal cancer cells. The activity of tumor cells can be inhibited by inducing apoptosis. Therefore, we hypothesize that Selumetinib may affect the mediator of NOLC1 and thus have therapeutic effects (Grasso et al. 2016). As a tyrosine kinase inhibitor, imatinib (IM) mainly acts through C-ABL, C-KIT, and platelet-derived growth factor (PDGF) receptor signaling pathways. Studies have found that imatinib blocking the C-KIT signal can reduce the expression of P-ELK1, thus reducing the LEVEL of CEA in colorectal cancer cells, and thus slowing down the progression of colorectal cancer. Lapatinib and cetuximab can be used as a reference for the treatment of KRAS wild-type, HER2-positive metastatic colorectal cancer in a long-term clinical trial (Ma et al. 2021a; Tosi et al. 2020).

In conclusion, NOLC1 may influence the occurrence and development of colorectal cancer through the above mentioned tumor-related signaling pathways, multi-gene regulation, tumor microenvironment, and other factors, and may have a potential clinical application value in improving the prognosis of colorectal cancer as a potential diagnostic and prognostic biomarker and therapeutic target. However, due to the limitations of this study, more basic experiments and large clinical trials are needed to verify the related biological functions and mechanisms of NOLC1.

## Data Availability

The data sets generated during and/or analysed during the current study are available from the corresponding author on reasonable request.
